# THE ASSOCIATION BETWEEN RACE, SEX, AGE, AND PERSONAL FINANCES DURING THE COVID-19 PANDEMIC

**DOI:** 10.1093/geroni/igae098.3734

**Published:** 2024-12-31

**Authors:** Lena Simon, Alexa Allan, Junyan Tian, Orfeu Buxton, Alan Zonderman, Michele Evans, Alyssa Gamaldo

**Affiliations:** Institute of Engaged Aging, Snellville, Georgia, United States; The Pennsylvania State University, University Park, Pennsylvania, United States; Penn State University, State College, Pennsylvania, United States; Pennsylvania State University, University Park, Pennsylvania, United States; National Institute on Aging, NIA, Maryland, United States; National Institute on Aging, NIA, Maryland, United States; Clemson University, Clemson, South Carolina, United States

## Abstract

The current study explored (a) the association between personal finances during the COVID-19 pandemic and depressive symptoms and (b) whether this association varies by sociodemographic characteristics. The sample included 158 aging adults (60% African Americans; 72% Female; age 46-83) from the Healthy Aging in Neighborhood of Diversity across the Life Span Sleep Study (HANDLSleep). Participants were asked to self-report changes in their finances during the COVID-19 pandemic on a scale from 0 (unstable or consistent decline) to 2 (consistent increase). The Patient Health Questionnaire-9 (PHQ-9) measured depressive symptomology and severity. Linear regression models indicated reports of unstable/consistent decline in finances during the COVID-19 pandemic were significantly associated with greater depressive symptomology even after adjusting for age, sex, and race (β = -.24, p =.034). Additionally, a significant interaction between race and COVID-19 finances item was observed (β =.91, p =.033). Nonsignificant interactions were observed between the COVID-10 finances item and other demographic variables (age, sex). Subsequent race-stratified analyses suggested that the significant association between unstable/consistent decline in finances during COVID-19 and greater depressive symptoms were observed only among White adults (β -.52, p =.008) but not within Black/African American adults (β = -.10, p >.05). These findings highlight the financial consequences of sociohistorical events (e.g., structural factors in a COVID-19 pandemic context) for mental health. These differential outcomes in groups of community dwelling adults suggest different public health measures and policies may be warranted under these circumstances.

